# The Urinary Hormonal State of Cats Associated With Social Interaction With Humans

**DOI:** 10.3389/fvets.2021.680843

**Published:** 2021-07-26

**Authors:** Takumi Nagasawa, Mitsuaki Ohta, Hidehiko Uchiyama

**Affiliations:** ^1^Department of Human and Animal-Plant Relationships, Graduate School of Agriculture, Tokyo University of Agriculture, Atsugi, Japan; ^2^Department of Animal Science and Biotechnology, Azabu University School of Veterinary Medicine, Sagamihara, Japan; ^3^Department of Human and Animal-Plant Relationships, Faculty of Agriculture, Tokyo University of Agriculture, Atsugi, Japan; ^4^Department of Animal Science, Faculty of Agriculture, Tokyo University of Agriculture, Atsugi, Japan

**Keywords:** cats, humans, social interaction, cortisol, oxytocin, urinary

## Abstract

Research to assess the relationship between cats and humans is in a nascent stage. Some studies have assessed the stress status in cats using physiological indicators, such as the cortisol hormone, but have not focused on the social interaction with humans. Moreover, the role of oxytocin secretion in the relationship between cats and humans remains unclear. In this study, we determined the possibility of quantifying the urinary concentration of oxytocin in cats and assessed the effects of social contact with humans on the levels of urinary oxytocin and cortisol metabolite. Four cats were subjected to two conditions, namely, social (control), and non-social (no social contact with humans) conditions. The levels of cortisol and oxytocin metabolite in urine samples from the cats in both conditions were determined using enzyme-linked immunosorbent assays. The urinary concentrations of cortisol and oxytocin under the non-social condition were significantly higher than those under the social condition. In addition, the concentration of oxytocin significantly correlated with that of cortisol in cats under the non-social condition. In this study, it was possible to quantify the concentration of oxytocin in the urine of cats, and the obtained results suggest that cats recognize the social interaction with humans as important. This information might contribute to the establishment of an assessment method for the welfare of cats and might help in clarifying the relationship between cats and humans.

## Introduction

The number of cats exceeds that of dogs in Japan (1), and this trend is common worldwide (2). The life expectancy of cats (3, 4) as well as of humans (5) has increased. To enhance the relationship between cats and humans, more information related to the human–cat interaction is needed.

There is a large and increasing number of questionnaires related to the human–animal interaction (HAI) (6). A questionnaire is an essential subjective indicator to assess the relationships between cats and humans. However, Rodriguez et al. (7) has highlighted the need to incorporate methodologically rigorous designs, combining both subjective and objective outcome measures, for developing the field of HAI. Thus, objective measurements, such as behavioral observation and physiological assessment, are also fundamental indicators in the field of HAI research. Behavioral observation, for example, the development of an ethogram (8, 9), is frequently used to assess the relationships between humans and cats. The cat stress score is a well-known observational assessment scale for evaluating the stress status in cats (10). Several studies on shelter cats used this assessment scale (11, 12); however, these studies have focused on the welfare of the cats, and not their relationship with humans.

Several physiological indicators, such as the heart rate variability and blood pressure, are mainly used in the field of veterinary research to assess the clinical conditions of cats (13, 14). Cortisol is a steroid hormone released to help cope with an acute stressor (15); it is, therefore, a useful indicator of the stress status in cats. Blood is a valid sample for quantifying the concentration of cortisol (16). However, procurement of this sample type is accompanied by physical confirmation (e.g., holding of the body) and an invasive procedure; thus, researchers should have confirmation sample types that can be collected non-invasively. For example, feces (17, 18) and hair (18) samples have been used to measure the concentration of cortisol. Especially in the field of HAI research, urine samples are useful because of the ease of collection. These studies have been conducted under various conditions, for example, in shelter (19, 20), laboratory (21), and house (22). Nevertheless, these studies were focused on the welfare of cats, not on their social interaction and relationship with humans.

Recently, oxytocin has received much attention in the field of HAI research. Oxytocin has variable functions, for example, in stress reduction, such as in decreasing the cortisol concentration and blood pressure (23), promoting well-being (24), and increasing social behavior (25). Rault et al. (26) mentioned that oxytocin is an essential indicator of psychological and social well-being in domesticated animals. Furthermore, oxytocin has a function related to pregnancy and uterine contractions (27), and is related to the construction of attachment relationships between infants and mothers (28). Some studies have shown relationships between dogs and humans, similar to those of infants and mothers (29).

Mutual interaction between dogs and their owners causes the secretion of oxytocin from their bodies (30, 31). Pet owners develop attachment not only with their dogs but also with cats (32). Therefore, oxytocin secretion may be a key factor in creating a bond between cats and humans. However, Potter and Mills (33) suggested that the attachment between cats and their owners is not transparent. Additionally, it is unclear whether oxytocin secretion is associated with the construction of a bond between cats and humans. To understand the genuine relationships between cats and humans, it is necessary to conduct a study focused on oxytocin. Blood oxytocin concentration in cats has been assessed by enzyme-linked immunosorbent assay (ELISA) (34). However, blood sampling can be quite stressful for the animals, and the evaluation of the correct physiological values is difficult. The content of urine is filtered from the blood, accumulated in a certain amount, and then naturally expelled. Therefore, urinary analysis may be an optimal non-invasive method for assessing these physiological conditions.

The purpose of this study was to determine whether the feline urinary cortisol and oxytocin metabolite concentrations could be quantified by ELISA. In addition, to clarify the relationship between cats and humans, we examined whether social contact with humans affects the concentration of these hormones in cats.

## Method

### Ethics Statement

The experiments performed in this study were approved by the Animal Experiment Ethics Committee (approval number: 1301312) at the Tokyo University of Agriculture in accordance with the World Medical Association's Declaration of Helsinki.

### Test Animals

The experiment was performed on four cats (A: 3-year-old, male, mix; B: 6-year-old, male; C: 10-year-old, female, Ragdoll; D: 3-year-old, female, mix). All the cats had always lived in a laboratory room (7 × 7 m) like a house cat. The cats freely spent time in the same room and were individually kept in a three-tier cage (93 × 63 × 178 cm) during the nighttime. Additionally, a caretaker looked after the cats as a house cat every day, ensuring proper feeding, physical care, playing, clicker training, physical contact (touching, petting, and grooming), and oral communication (calling and talking).

### Assay Methods

#### Collection of Urine Samples

To ensure the welfare of the cats, we adopted a non-invasive method of urine collection by natural urination. Additionally, we collected spot urine samples, instead of pooled samples, each time from the tray in litter boxes and transferred them directly to plastic 2-ml centrifuge tubes. The samples were kept frozen at −80°C until analysis. For quantification, the supernatant obtained after centrifugation of the urine samples at 1,661 × *g* for 15 min at 4°C was used.

#### Quantification of Cortisol in Urine Samples

Urinary cortisol metabolite concentration was determined using the DetectX® Cortisol Enzyme Immunoassay Kit (K003—H5W, Arbor Assays LLC, USA; goat anti-mouse IgG) used in previous studies (35, 36). The assay standard curve ranged from 50 to 3,200 pg/ml, and the assay sensitivity was 27.6 pg/ml. The urine samples were diluted 10-fold with the assay buffer. The intra-assay coefficient of variation (CV) for the cortisol assay was 4.10%, and the inter-assay CV was 5.25%.

#### Quantification of Oxytocin in Urine Samples

The urine samples were extracted with a Hyper Sep C18 column (3 ml/200 g, Thermo Fisher Scientific, Tokyo), as described by Finkenwirth et al. (37). Previous studies successfully quantified the urinary metabolite concentrations in dogs, wolves, and humans (38, 39). The C18 column was conditioned by washing three times with 3 ml of 100% methanol and then three times with 3 ml of distilled water. A mixture of 1 ml of urine sample and 10 μl of phosphoric acid was transferred to the column. The columns were washed with 3 ml of 10% acetonitrile and 0.1% trifluoroacetic acid. Samples were then eluted with 1 ml of 80% acetonitrile. The eluted samples were dried using an evaporator. The dried samples were reconstituted in 1 ml of assay buffer provided with the kit and used for determining the metabolite concentration of oxytocin. Urinary oxytocin metabolite concentration was determined using the DetectX® oxytocin Enzyme Immunoassay Kit (K048 - H5, Arbor Assays LLC; goat anti-rabbit IgG) used in previous studies (40, 41). The assay standard curve ranged from 16.38 to 10,000 pg/ml, the assay sensitivity was 17.0 pg/ml, the intra-assay CV for the oxytocin assay was 4.25%, and the inter-assay CV was 4.59%.

#### Quantification of Creatinine in Urine Samples

Throughout the experiments, the four cats had free access to drinking water; thus, the water intake of individual cats varied daily. Therefore, the urinary hormone concentrations need to be corrected by urinary creatinine to account for the quantity of water in the sample. All oxytocin and cortisol levels were described as pg/mg creatinine (Cre). The concentration of creatinine was measured by the Jaffe reaction using 96-well microplates (3881–096, Iwaki, Japan). After the reaction, the optical density was read at 490 nm using a microplate reader.

### Experimental Protocol

The experiment was performed under two conditions: social condition (SC) and non-social condition (NSC). In SC, urine samples were continuously collected for 3 days. In NSC, arrangements were made such that the caretaker engaged in minimum necessary care (e.g., feeding and managing the environment), excluding social contact (e.g., physical care, playing, clicker training, physical contact, and oral communication) for 3 days. The interval between the experiments under the two conditions was 2 weeks. All cats were fed a constant amount of food throughout the experiment to negate its effects on urinary hormone metabolite concentrations.

### Statistical Analyses

We excluded outliers, defined as 1.5 × interquartile range (IQR), from the analysis. The difference in the mean hormone concentration during SC and NSC was determined using the Welch's *t*-test or Mann–Whitney *U*-test. The effect sizes were calculated by Cohen's d. Using Spearman's rank correlation coefficient, we assessed the correlation between cortisol and oxytocin concentrations. Statistical significance was set at *p* < 0.05. All statistical analyses were performed using BellCurve for Excel (Social Survey Research Information Co., Ltd., Japan). There was no outliner in the cortisol assay, whereas there were three outliers in both SC and NSC in the oxytocin assay.

## Results

**Figure 1:**
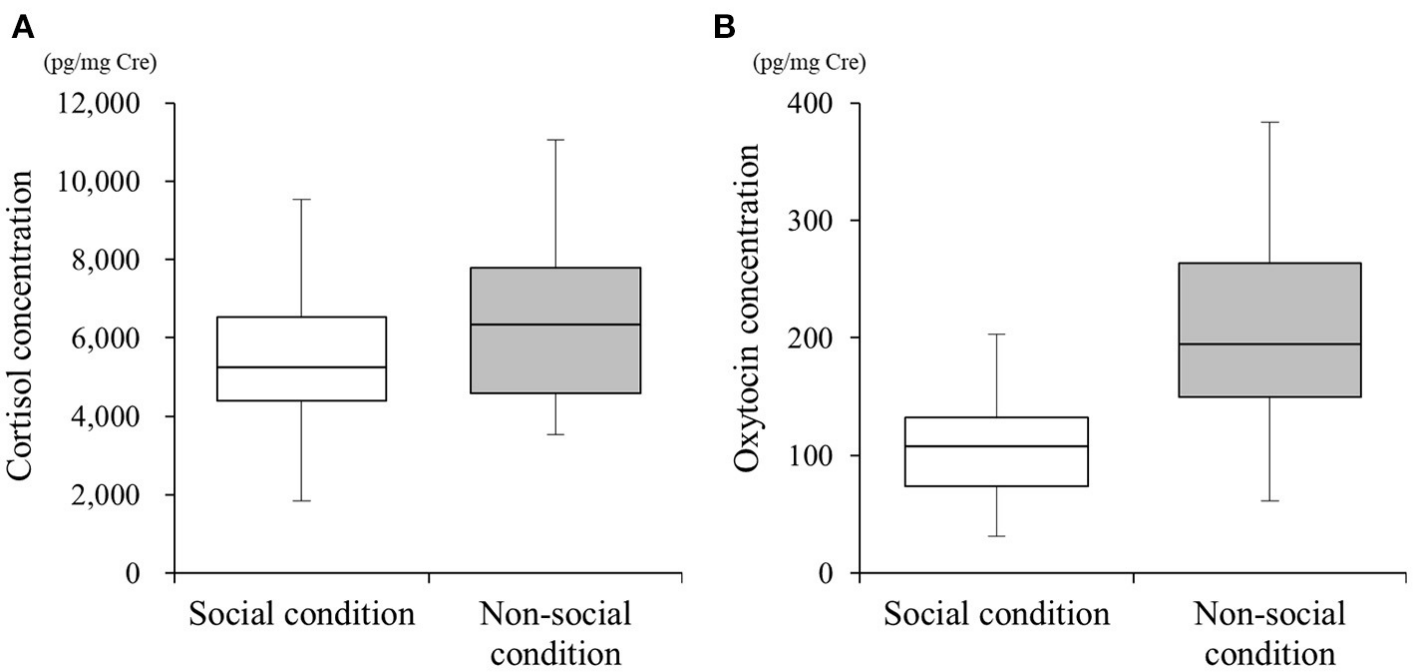
Differences in urinary cortisol **(A)** and oxytocin **(B)** concentrations under social (SC) and non-social (NSC) conditions. Box plots show the interquartile range (IQR) for each condition, with whiskers extending to 1.5 × the IQR.

We collected 54 urine samples and performed the different quantification assays. The mean cortisol metabolite concentrations in SC and NSC were 5,433.40 ± 1,805.02 (*n* = 25, range 1,843.53–9,528.73) and 6,339.98 ± 1,908.95 (*n* = 29, range 3,531.81–11,048.50) pg/mg•Cre, respectively (Figure 1A). The mean oxytocin metabolite concentrations in SC and NSC were 115.72 ± 9.28 (*n* = 22, range 31.62–203.48) and 193.06 ± 82.57 (*n* = 26, range 61.63–383.91) pg/mg•Cre, respectively (Figure 1B).

**Figure 2:**
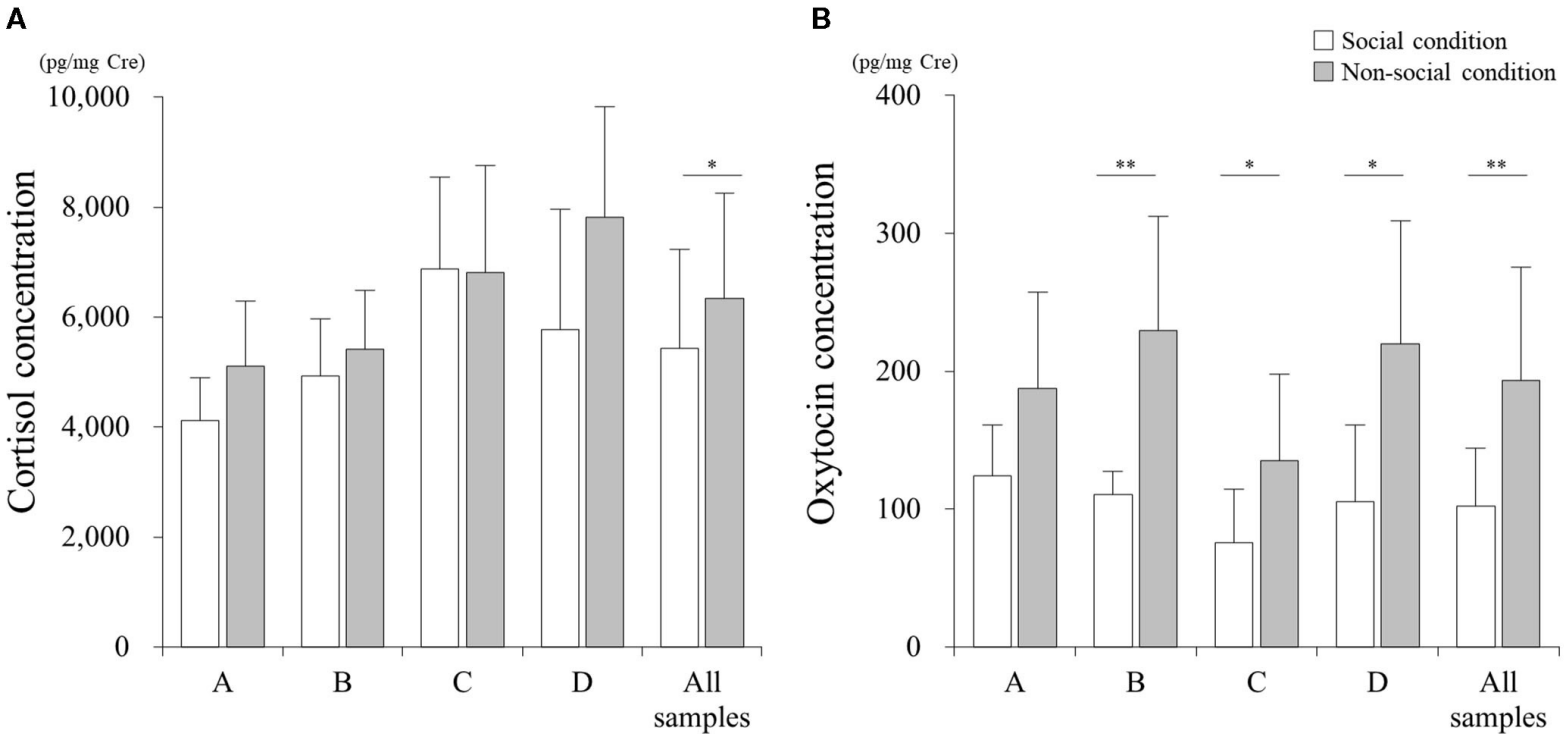
Comparisons of cortisol **(A)** and oxytocin **(B)** concentrations between social (SC) and non-social (NSC) conditions. *p < 0.05, **p < 0.01.

There was a significant difference in the cortisol concentration between SC and NSC conditions in all 54 samples (Figure 2A, *p* < 0.05; Cohen's d: 0.49). However, no differences were found when analyzing the samples collected from each individual: A (*p* > 0.05; Cohen's d: 0.98), B (*p* > 0.05; Cohen's d: 0.44), C (*p* > 0.05; Cohen's d: 0.02), and D (*p* > 0.05; Cohen's d: 0.96).

The concentration of oxytocin in NSC significantly increased relative to that in SC for all the samples (Figure 2B, *p* < 0.01; Cohen's d: 1.39), and samples belonging to cats B (*p* < 0.01; Cohen's d: 1.98), C (*p* < 0.05; Cohen's d: 1.14), and D (*p* < 0.05; Cohen's d: 1.54); however, there was no difference for cat A (*p* > 0.05; Cohen's d: 1.13).

**Figure 3:**
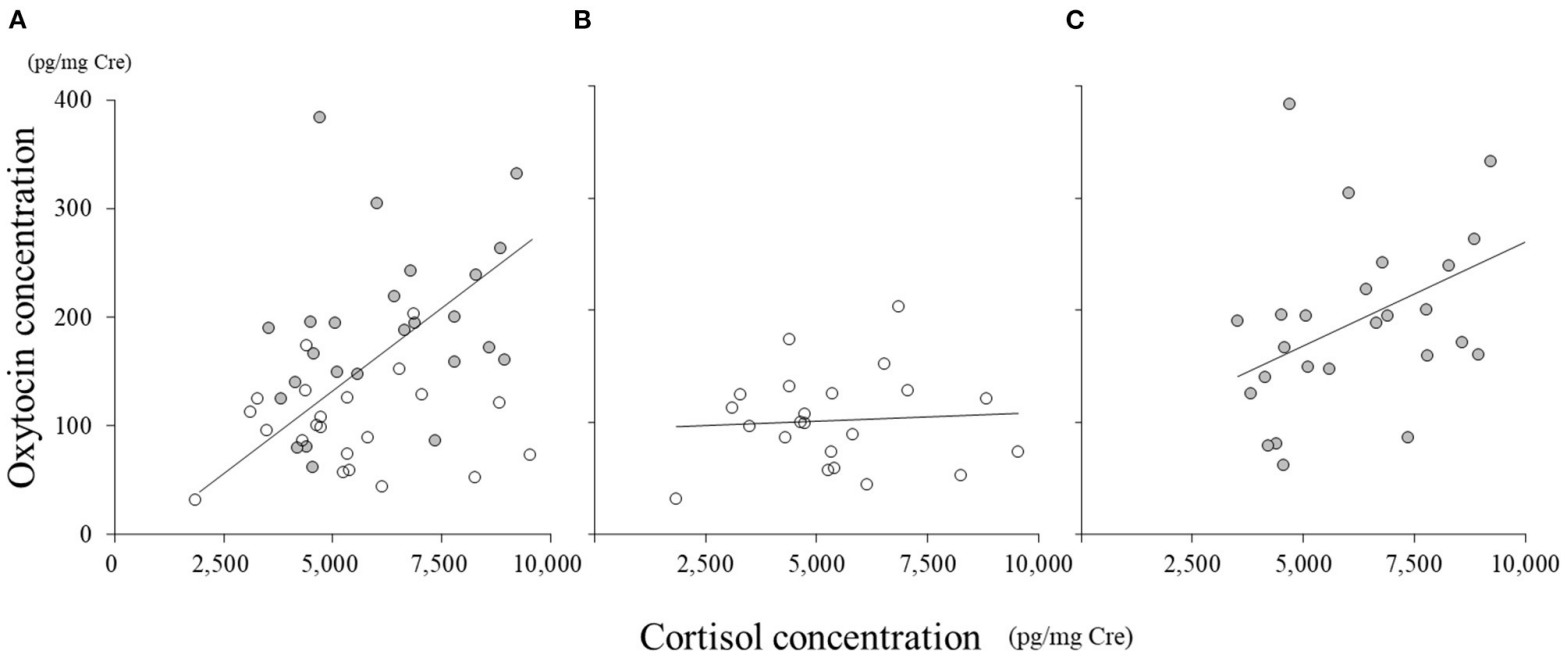
Correlations between oxytocin and cortisol concentrations. **(A)** All samples; **(B)** social condition (SC); **(C)** non-social condition (NSC).

In all the samples, there was a significant correlation between cortisol and oxytocin concentrations (Figure 3A, *r* = 0.40, *p* < 0.01). In SC, the concentration of cortisol did not correlate with that of oxytocin (Figure 3B, *r* = 0.07); however, in NSC, cortisol concentration significantly correlated with that of oxytocin (Figure 3C, *r* = 0.45, *p* < 0.01).

## Discussion

We showed that it is possible to quantify the urinary metabolite concentrations of hormones in cats using ELISA; hitherto, there has only been one report (34) on the quantification of the blood oxytocin concentration in cats. Measurement of both cortisol and oxytocin might help in accurately assessing the physiological status of cats because oxytocin influences the activities of the hypothalamic–pituitary–adrenal axis and autonomic nervous system, as does cortisol (23). Measurement of not only cortisol but also of oxytocin is a useful method to accurately understand the physical status of house cats; therefore, our results are of great importance in the field of HAI.

It is notable that we used the natural spot of urination of cats. Because urine sampling through an invasive method using catheters might have negative effects on the welfare of cats, we adopted a non-invasive method. Thus, methods for collection of samples for physiological assessment of cats under different situations (e.g., laboratory cats, shelter cats, stray cats, and household cats) should be expanded. Additionally, in this study, we focused on urinary metabolite concentration, and not locally produced and circulating hormones. Urine metabolites accumulate in the bladder for a long time; thus, urinary metabolite concentration reflects the long-term physiological condition. Urinary metabolite hormone concentrations correlate with circulating hormone concentration (42). This is an advantage in assessing the basal and long-term physiological states, and not just temporary and short-term states. The purpose of this study was to determine the physiological state of cats for 3 days; therefore, metabolite concentrations served as reasonable indicators. Future studies should focus on developing quantification methods for urinary hormone metabolites as assessment tools for the welfare of cats.

There is controversy regarding the immune-reactivity of urinary oxytocin metabolite in ELISA. Previous studies in dogs, wolves, and humans reported that urinary oxytocin metabolite has two peaks of immune-reactivity although the oxytocin metabolite concentration can be quantified (38, 39, 42). Moreover, a previous study reported no relationship between OT in the plasma and urinary samples in humans (43). The findings of this study should be interpreted carefully. In the future, it is necessary to verify the immune reactivity of urinary oxytocin in cats.

In the present study, the metabolite concentrations of both cortisol and oxytocin under NSC were higher than those under SC. Acute stressors induce the secretion of cortisol; thus, cats might perceive the interception of social communication with humans as a stressful event. In previous studies, interaction with humans has positive physiological and behavioral effects on shelter cats (44, 45). Our results possibly support the results of these studies from the perspective of hormonal change. Additionally, oxytocin concentrations in three out of four cats were different between SC and NSC conditions, although the difference for cortisol concentrations was not confirmed. Oxytocin has the functions not only to inhibit and reduce stress (46, 47) but also to promotes social behavior (48–50). Oxytocin might have been secreted in cats seeking social interaction with humans; therefore, we believe that cats recognize interactions with humans as important. Moreover, in NSC, but not in SC, urinary cortisol concentration was significantly correlated with oxytocin. The basal plasma concentrations of oxytocin and cortisol have a positive correlation only when the experimental situation causes anticipation of stress or a novel situation (51). Our results show that cats might have perceived NSC as a stressful event, and as such, the concentration of urinary oxytocin would be correlated with that of urinary cortisol under social stress conditions, but not under normal conditions.

It is not easy to interpret the results of this study. At first, oxytocin has variable physiological functions; thus, the rigorous causal relationship for consequence is still unclear. Second, there is evidence showing the negative correlation between circulating oxytocin and cortisol (52), namely, oxytocin has the function to inhibit the activity of cortisol. The reverse consequence of this study may be explained as a difference in the period of interest. The phenomenon that oxytocin decreases the cortisol concentration might occur following both cortisol and oxytocin temporal increase. In the case of the result of the above whole phenomenon, both the urinary oxytocin and cortisol metabolite concentration might be high levels; therefore, it is a possible explanation that both urinary oxytocin and cortisol of cats showed high levels in the social stress condition. However, it is very difficult to judge the conclusion by only this study. This study had a potential limitation, as only four cats were included in this study, and the number of urinary samples collected was small. Moreover, the cats were only kept for 3 days under NSC considering the welfare of the cats. In the future, it is required that more cats and urine samples are analyzed, and deeply discuss the relationship between cortisol and oxytocin.

Herein, we demonstrated the possibility of quantifying the urinary metabolite concentration of oxytocin in cats. Moreover, the urinary metabolite concentrations of oxytocin and cortisol in cats were found to be influenced by social interaction with humans. The results of the present study should contribute to the development of strategies for the welfare of cats and should provide a new perspective on the social relationship between cats and humans.

## Data Availability Statement

The original contributions presented in the study are included in the article/supplementary material, further inquiries can be directed to the corresponding authors.

## Ethics Statement

The animal study was reviewed and approved by Animal Experiment Ethics Committee at the Tokyo University of Agriculture.

## Author Contributions

TN performed the experiments and analyzed the data. All authors planned the experiments and wrote the manuscript.

## Conflict of Interest

The authors declare that the research was conducted in the absence of any commercial or financial relationships that could be construed as a potential conflict of interest.

## Publisher's Note

All claims expressed in this article are solely those of the authors and do not necessarily represent those of their affiliated organizations, or those of the publisher, the editors and the reviewers. Any product that may be evaluated in this article, or claim that may be made by its manufacturer, is not guaranteed or endorsed by the publisher.
